# Male reproductive phenotype in cystic fibrosis: comparison of existing animal models

**DOI:** 10.1038/s41684-026-01763-0

**Published:** 2026-06-30

**Authors:** Angèle Boursier, Charles-Henry Cottart, Aissatu Balde-Camara, François Vialard, Anne-Laure Barbotin

**Affiliations:** 1 Institut de Biologie de La Reproduction-Spermiologie-CECOS, Hôpital Jeanne de Flandre, CHU Lille, Lille, France; 2 U1172 - LilNCog - Lille Neuroscience & Cognition, Univ. Lille, INSERM, CHU Lille, Lille, France; 3 Service de Biochimie, Hôpital Necker-Enfants Malades - Assistance Publique Hôpitaux de Paris, Paris, France; 4 INSERM 1151, INEM Université de Paris Cité, Paris, France; 5 Physiologie Humaine et Expérimentale, Faculté de Pharmacie - Université de Paris Cité, Paris, France; 6 INSERM UMR 1357 IMPROVE, UVSQ, Versailles, France

**Keywords:** Urogenital reproductive disorders, Infertility

## Abstract

In men, cystic fibrosis (CF) leads to infertility in over 95% of cases, due to early and progressive atresia of the vas deferens, resulting in obstructive azoospermia. The advent of highly effective modulator therapy has enabled more men with CF to consider parenthood, raising important questions regarding the impact of these treatments on male fertility. Animal models provide major insights into the mechanism of CF-related reproductive tract abnormalities, yet high interspecies variability complicates model selection. Mouse models display heterogeneous phenotype: knock-in or partial knockout models usually remain fertile, whereas complete knockouts may develop vas deferens atresia with aging. Among currently studied models, CF transmembrane conductance regulator (CFTR)-knockout rats more closely reproduce the human phenotype, showing bilateral absence of the vas deferens and epididymal hypoplasia, although they exhibit more pronounced hypospermatogenesis than observed in men. In larger animal models, including ferrets, pigs, sheep and rabbits, absence of the vas deferens duct and epididymis is frequently observed, often with normal testicular histology; however, most studies in these species have mainly focused on neonatal stages, leaving long-term reproductive outcomes uncharacterized. No single model fully replicates human male reproductive pathology in CF. Combining rodent and large-animal models is probably essential to elucidate the mechanisms of vas deferens absence and to assess the long-term effects of CFTR modulators on male spermatogenesis and reproductive outcomes.

## Abstract (web summary)

This Review discusses how animal models recapitulate male infertility in cystic fibrosis and highlights the need for combined rodent and large-animal studies to investigate reproductive pathology and the effects of highly effective modulator therapy.

## Main

Cystic fibrosis (CF) is an autosomal recessive disorder caused by mutations in the *CFTR* gene, which encodes the CF transmembrane conductance regulator (CFTR) protein, an anion channel responsible for chloride and bicarbonate ion transport across epithelial cell membranes^1^. CFTR is widely expressed in various organs, including the lungs, pancreas, intestines and reproductive tract. In men with CF, impaired or absent CFTR function leads to the accumulation of abnormally thick mucus in the affected organs. This accumulation results in chronic inflammation and progressive tissue damage, which are hallmark features of CF pathophysiology^2^.

The male reproductive tract is particularly sensitive to CFTR dysfunction. More than 95% of men with CF present with bilateral absence of the vas deferens^3^. Current evidence suggests that CFTR-related absence of the vas deferens most often results from progressive obstruction and secondary degeneration of the vas deferens during fetal life rather than true congenital agenesis^4^.

These alterations result in a complete absence of spermatozoa in the ejaculate (that is, obstructive azoospermia), precluding natural conception. When men with CF wish to conceive, sperm can be surgically retrieved (testicular or epididymal extraction) and used for assisted reproductive technologies, which offer favorable outcomes in this context^5,6^. In rare cases, men with CF present with intact vas deferens. This phenotype is more commonly associated with mild CF-causing variants, such as the 3849 + 10 kb C → T splice variant, whereas other variants, such as R117H, are strongly associated with bilateral absence of the vas deferens^7^. A French study reported that one-fourth of 48 men with CF who achieved biological fatherhood did so through natural conception, with the proportion being significantly lower among those carrying severe CF genotypes than among those carrying mild variants. However, this study did not include data confirming the paternity of these natural conceptions (that is, absence of genetic confirmation of biological fatherhood)^8^.

In humans, CFTR is highly expressed in the fetal male reproductive tract, notably in Wolffian-derived structures, such as the rete testis, epididymis and vas deferens^4^. CFTR is also highly expressed in the efferent ducts, which derive from the mesonephric tubules^9,10^. Although these structures have not been specifically described in patients with CF, their role in fluid reabsorption and sperm concentration suggests that functional impairment in CF cannot be excluded.

Bilateral absence of the vas deferens in CF is associated with absence of the seminal vesicles in approximately 50% of cases and less consistently with epididymal anomalies^11,12^. The underlying mechanism is believed to involve progressive obstruction and atresia of the vas deferens due to abnormal intraluminal secretions. Fetal studies have demonstrated a morphologically normal reproductive tract up to 22 weeks of gestation, followed by lymphoid infiltrates, epithelial destruction and mucinous plugs from 26 weeks onward, consistent with a secondary degenerative process^3,4^. Ultrasound studies in children with CF further support this theory, as pediatric patients exhibit fewer urogenital tract anomalies than adults^13^.

Spermatogenesis is maintained at least at a moderate level in men with CF, although some studies have reported lower testosterone levels, suggesting possible testicular involvement^14^. In a 2025 publication, we reported lower rates of epididymal sperm retrieval in patients without CFTR residual activity compared with those with residual activity, suggesting testicular impairment in the absence of CFTR function^6^.

The advent of CFTR modulators has transformed the clinical landscape and substantially improved quality of life for patients with CF, enabling more individuals to consider and pursue parenthood. Consequently, fertility has become a central concern in CF management. The widespread use of highly effective modulator therapy raises new questions regarding its potential impact on reproductive health. Indeed, early use of CFTR modulators may have implications for male reproductive outcomes^15^. Because CFTR modulators improve mucus clearance and are administered earlier in life, including during pregnancy and early childhood, the key question is whether these treatments can preserve the vas deferens and epididymal structures, possibly by promoting the fluidification of intraluminal secretions. This possibility is supported by two recent case reports describing clinical outcomes in boys exposed in utero to elexacaftor/tezacaftor/ivacaftor. In the first report, deferential structures appeared normal in a boy at 2 weeks and 3 months of age^16^, whereas in the second report, normal reproductive anatomy was observed in a boy at 8 weeks and 6 months of age^17^. Because placental transfer of CFTR modulators has been documented, questions remain regarding the potential long-term consequences of an early and prolonged exposure to CFTR modulators, which may begin as early as the prenatal or neonatal period^18^. While these therapies may support the preservation of the epididymis and vas deferens, their long-term effects on testicular development and spermatogenesis remain to be elucidated.

In this context, evaluation of male reproductive outcomes across existing CF animal models is essential. Multiple animal models of CF are available, showing interspecies variability, including in the reproductive tract^19^. Comparative analysis of reproductive tract abnormalities and fertility endpoints is critical to identify the models that most accurately replicate the human phenotype and are best suited for studies on male fertility in CF. The aim of this Review is therefore to compare reproductive phenotypes across male CF animal models, highlight differences from human pathophysiology and identify the most relevant models for studying male fertility in CF.

## Mouse models

Mouse models of CF were the first to be generated, in 1992, with the development of CFTR knockout (KO) animals^20^. So far, at least 15 CF mouse models have been described and can be classified into three main categories: (1) CFTR KO models, (2) CFTR models with residual function and (3) models carrying mutations that reproduce human CFTR variants^21^. Although the high degree of homology between human and murine CFTR suggested that the mouse could provide a reliable CF model^22^, the absence of typical pulmonary and pancreatic alterations in mouse models has substantially limited their use^19^.

In contrast to other organs, male reproductive features and fertility outcomes have rarely been studied in mouse models, and reported findings are inconsistent. Discrepancies exist regarding both the presence and age-dependent evolution of vas deferens and epididymal alterations. Table 1 summarizes the reported reproductive tract abnormalities and fertility outcomes across the different mouse models.

**Table 1:** Summary of male reproductive phenotypes across animal models of CF

Species	Genetic model		Genetic background and/or strain	Ref.	Animals’ age	Fertility status of homozygous males	Macroscopic reproductive tract defects	Histological abnormalities	Semen parameters
Mouse	*Cftr*^*tm1Unc*^ (CFTR KO)		Mixed (129/Ola × C57BL/6 × DBA/2 × BALB/c)	^20^	30–40 days	Fertile (2 males reaching maturity)	None	None	NE
			Congenic C57BL/6J	^33^	3–15 months	Infertile (*n* not specified)	8 out of 10 animals without VD and 2 males between 3 and 5 months with VD	Reduced VD lumen diameter and thickened smooth muscle layer	Reduced sperm count in E and fewer late spermatids in ST
			Mixed (129/Ola × C57BL/6 × DBA/2 × BALB/c)	^34^	2–6 months	NE	NE	Slight reduction in ST diameter	Decreased expression of protamin-2, a marker of spermatid and spermatozoa
			Mixed (129/Ola × C57BL/6 × DBA/2 × BALB/c)	^31^	Days 21, 40–60 and 80–100	Liquid-fed males (*n* not specified): infertile; solid-fed males: fertile (2 out of 3)	None (at any age)	NE	NE
	*Cftr*^*tm1Cam*^ (CFTR KO)		Mixed 129 × C57BL-6	^28^	Days 10, 15, 20, 40, 44, 50, 80 and 100	Fertile (1 male out of 3 tested)	None	Normal until day 40; smaller VD lumen with intraluminal secretions at later stages	No sperm in VD at days 40 and 44; sperm detected in males aged 100 days
	*Cftr*^*tm3Bay*^ (CFTR KO)		Mixed 129/Sv × C57BL/6J	^32^	Not specified (adults)	Fertile (*n* not specified)	NE	NE	NE
	*Cftr*^*tm1Bay*^ (CFTR KO with residual function)		Mixed 129/Sv × C57BL/6J	^29^	3–4 weeks and 6 weeks	Fertile (6 males of 6 weeks)	None (5 males of 3–4 weeks and one 6 weeks old)	NE	NE
	*Cftr*^*tm1Hgu*^ (CFTR KO with residual function)		Mixed 129/Ola × C57BL/6J	^30^	30 days	NE	None	1 out of 6 males showed increased mucin in VD	NE
	*Cftr*^*tm1Eur*^ (CFTR-F508del)		Mixed 129/Ola × FVB	^24^	5–7 weeks	NE	None	No mucus accumulation in VD	NE
			Mixed 129 × FVB	^28^	Days 10, 15, 20, 40, 44, 50, 80 and 100	Fertile (1 male out of 1 tested)	None	Normal until day 40; smaller VD lumen with intraluminal secretions at later stages	No sperm in VD at days 40 and 44; sperm detected in males from 50 days onward
	*Cftr*^*tm1Kth*^ (CFTR-F508del)		Mixed 129/Ola × C57BL/6J × FVB/N	^25^	Not specified (young adults)	Fertile (*n* not specified)	None	NE	NE
	*Cftr*^*tm1G551D*^ (CFTR-G551D)		Mixed 129/Sv × CD-1	^26^	Not specified (adults)	NE	None	NE	NE
	*Cftr*^*tm2Hgu*^ (CFTR-G480C)		Mixed 129/Ola × C57BL/6J	^27^	Not specified (adults)	Fertile (*n* not specified)	NE	NE	NE
Rat	CFTR KO (CRISPR–Cas9, SD)	SD		^38^	Young adults ≤6 months)	NE	Absence of VD	NE	NE
				^37^	8–12 weeks	NE	VD, E and SV hypoplasia	Hypospermatogenesis with reduced diameter of ST, multinucleated giant cells in the lumina of ST (spermatid coalescence)	Complete absence of cauda epididymal sperm
	CFTR KO (ZFN)	SD		^36^	6 weeks	NE	Absence of VD	NE	NE
	CFTR-F508del (CRISPR–Cas9)	SD		^37^	8–12 weeks	NE	VD, E and SV hypoplasia	Hypospermatogenesis with reduced diameter of ST; multinucleated giant cells in the lumina of ST (spermatid coalescence)	Normal to moderate epididymal sperm levels; morphological abnormalities with head–tail separation
				^38^	Young adults (≤6 months)	NE	Absence of one or two VD or normal phenotype	NE	NE
Rabbit	CFΔ1 (CFTR KO, CRISPR–Cas9)	NZW		^39^	300 days	Infertile (*n* = 4)	Absence of VD and E	NE	NE
Ferret	CFTR KO (AAV)			^41^	Neonates (birth)	NE	VD absent or segmentally absent	Degenerate to tortuous and serpentine VD epithelium	NE
	*Cftr*^*G551D*^ (AAV)			^42^	Neonates (birth)	NE	Absence of VD and E	NE	NE
Sheep	CFTR KO (CRISPR–Cas9)			^43^	0–3 days	NE	Absence of VD, E mostly present	Normal testis histology	NE
Pig	CFTR KO (AAV)			^44^	0–2 days	NE	None (*n* = 20)	NE	NE
				^45^	Neonates (birth)	NE	Partial absence of VD and/or E in 32/34; rudimentary caput E present; normal testis	Fibrous tissue replacing VD; smooth muscle disorganized	NE
	CFTR-F508del (AAV)			^45^	Neonates (birth)	NE	Partial absence of VD and/or E in 17/19; sometimes complete VD absence; rudimentary caput E present; normal testis	Fibrous tissue replacing VD; smooth muscle disorganized	NE
	CFTR KO (BAC targeting)			^46^	Neonates (birth)	NE	VD absent or dysplastic; rudimentary caput E present	Connective tissue instead of tubular profiles in the caudal E	NE

Animal models lacking published data on male reproductive phenotype were not included. NE, not evaluated; VD, vas deferens; E, epididymis; ST, seminiferous tubules; SV, seminal vesicles; ZFN, zinc-finger nucleases; SD, Sprague-Dawley rats; NZW, New Zealand White rabbits; AAV, adeno-associated virus.

It is noteworthy that structural differences exist between the human epididymis and rodent models. In rodents, the proximal epididymis contains a well-differentiated initial segment. By contrast, the human epididymis lacks a clearly defined initial segment; its proximal region is formed in continuity with the efferent ducts and shows less pronounced compartmentalization. Furthermore, rodents exhibit a well-developed distal cauda epididymis with substantial sperm reservoir capacity. While the intraluminal diameter increases along the epididymal tubule in rodents, the human epididymal lumen remains relatively small throughout the organ, possibly contributing to the faster sperm transit observed in humans (reviewed in ref. ^23^).

Male mice carrying human CFTR mutations (F508del, G551D or G480C) are generally fertile, with intact epididymides and vas deferens^24–28^. In the study by Reynaert et al.^28^, the timing of sperm appearance in the vas deferens was delayed in mutant animals: spermatozoa were detected from day 40 after birth in wild-type animals, but only from day 50 in CFTR-F508del mice, with persistence at later stages. The study also showed that vas deferens lumina in these mutants were normal up to day 40. After that, the luminal diameter decreased and secretions accumulated, although no additional structural differences compared with wild-type animals were reported^28^.

Regarding CFTR models with residual function, they do not exhibit macroscopic reproductive tract abnormalities or infertility^29,30^. In one study, only a single *Cftr*^*tm1Hgu*^ male mouse was found to have increased mucin content in the vas deferens^30^.

By contrast, complete CFTR KO models display heterogeneous and age-dependent reproductive phenotypes. While several studies have reported fertile males without macroscopic or histological alterations of the reproductive tract^20,31,32^, others, most notably those investigating older (3–15 months) *Cftr*^*tm1Unc*^ mice bred to congenic C57BL/6J, describe infertility, with bilateral absence of the vas deferens in 8/10 males; vas deferens was visible in only 2/10 males aged 3–5 months^33^. Histology and semen analysis in these older mutant animals revealed reduced epididymal sperm counts and fewer late spermatids in the seminiferous tubules compared with wild-type animals^33^. Studies extending to 6 months in CFTR KO animals with mixed genetic background also reported reduced seminiferous tubule diameter and decreased expression of protamine-2, a specific marker of spermatids and spermatozoa^34^.

Overall, most investigations have been conducted in mice younger than 3 months, while reproductive tract alterations seem to emerge at later stages in complete CFTR KO models.

Finally, some investigators have suggested a delay in puberty in CF mice, presumably because of their reduced body weight^35^, which is consistent with the delayed appearance of sperm in the vas deferens reported in both F508del and CFTR KO mice^28^.

## Rat models

The first CFTR KO rat model was generated in 2014^36,37^ and subsequent F508del knock-in models have since been developed^37,38^. Rat models more closely recapitulate key features of human CF, including some aspects of pulmonary disease and pancreatic involvement^21^. They represent a more physiologically relevant alternative to mouse models, combining improved disease fidelity with ease of handling and relatively affordable housing costs.

Male reproductive phenotypes in CFTR KO rats seem to be closer to the human CF phenotype, as adult animals constantly exhibit bilateral absence or hypoplasia of the vas deferens^36–38^. McCarron et al.^37^ additionally reported hypoplasia of the epididymis and seminal vesicles, along with testicular histological alterations consistent with hypospermatogenesis (that is, reduced testicular sperm production). These alterations included decreased seminiferous tubule diameter, disorganization of the seminiferous epithelium and a reduced number of germ cells, particularly mature spermatids, in CF animals. Consequently, no sperm were detected in the cauda epididymis of these animals^37^. A milder degree of testicular dysfunction is suspected in patients with CF, although it has not yet been formally demonstrated. Although breeding of CFTR KO male rats has not been attempted, they are probably infertile due the combined absence of vas deferens and defective spermatogenesis.

F508del rats display a more heterogeneous phenotype: Dreano et al.^38^ reported variable presence of one or both vas deferens, although the frequency of each phenotype remains unknown. McCarron et al.^37^ observed that F508del rats display a phenotype similar to that of CFTR KO rats, with hypoplasia of the vas deferens and epididymis as well as hypospermatogenesis. However, in contrast to CFTR KO males, F508del rats exhibited normal to moderately reduced epididymal sperm counts in the cauda epididymis, although sperm cells exhibited morphological abnormalities such as head–tail disjunction^37^. This discrepancy between F508del and CFTR KO rats may be explained by residual CFTR function in F508del animals, as demonstrated by Dreano et al.^38^, which could mitigate the severity of reproductive tract abnormalities.

## Rabbit models

The first CFTR KO (CFΔ1) rabbit model of CF was established in 2021 by Xu et al.^39^. These animals exhibited some manifestations of CF, such as gastrointestinal obstruction and growth restriction, but they did not spontaneously develop lung disease. Four adult males were reported to be infertile and exhibited a complete absence of both vas deferens and epididymis. No further investigations were conducted regarding testicular histology or spermatogenesis^39^.

## Ferret models

In 2008, Sun et al. developed the first CFTR KO ferret, which reproduced key features of the human pathology, including ileus meconium, pancreatic involvement and early airway inflammation^40^. In a cohort of five male kits, one male exhibited complete absence of the vas deferens, while the remaining animals showed segmental or unilateral absence of the deferens. By contrast, heterozygous *CFTR*^+/–^ kits had consistently normal vas deferens^41^. At that kit stage, epididymal morphology, spermatogenesis, histopathological abnormalities and long-term evolution of deferential structures were not studied. Another study from the same group confirmed these findings, reporting the absence of epididymis and vas deferens in four CFTR KO kits^42^.

The team also generated a CFTR-G551D knock-in model and conducted a detailed investigation of the CFTR-G551D male reproductive tract. *CFTR*^G551D^ homozygotes displayed the same phenotype as KO kits, characterized by the absence of epididymis and vas deferens (observed in three CFTR-G551D kits). Remarkably, when pregnant jills were treated with VX-770 (ivacaftor), their *CFTR*^*G551D/G551D*^ offspring were born with both epididymal and deferential structures^42^. This report represents the first documented case of in utero vas deferens preservation by CFTR modulator therapy in animal models.

## Sheep models

The first CFTR KO sheep model of CF was developed by Fan et al.^43^ in 2018 and was considered relevant due to the anatomical and developmental similarities between the ovine and the human lungs. Histological assessment of 12 *CFTR*^−/−^ newborn lambs revealed absence of the vas deferens, while the epididymis was grossly evident. By contrast, three heterozygous males had normal reproductive ducts. Testicular histology was reported to be normal in all lambs examined^43^. So far, fertility outcomes in adult males have not been evaluated in this model.

## Pig models

In 2008, Rogers et al.^44^ developed the first CFTR KO pig model, leveraging the anatomical similarities between porcine and human airways to address the limitations of mouse models, which fail to develop spontaneous lung disease. Initial observations in 20 *CFTR*^−/−^ piglets suggested that the vas deferens was normal at birth^44^. Subsequent studies, however, demonstrated that the majority of CFTR KO piglets exhibited absence of the vas deferens and epididymis, characterized by rudimentary caput epididymides with reduced tubular structures, while the testes remained normal^45^. A second CFTR KO pig model generated using bacterial artificial chromosomes (BAC) vectors displayed a similar phenotype, with absent or hypoplastic vas deferens and a rudimentary caput epididymis with a reduced ductular network^46^.

Evaluation of the *CFTR*^*F508del/F508del*^ model revealed a highly similar pattern of male reproductive tract abnormalities, with 17 of 19 piglets showing absence or hypoplasia of the vas deferens and an isolated rudimentary caput epididymis^45^.

Interestingly, when studying the potential mechanisms underlying the absence of vas deferens and epididymis in the CFTR KO and CFTR-F508del pig models, Pierucci-Alves et al.^45^ found no differences in mucus accumulation within the reproductive tract compared with wild-type pigs, suggesting that the reproductive tract abnormalities develop independently of mucus inspissation or luminal obstruction. Proposed contributing mechanisms include (1) enhanced transforming growth factor-β (TGFβ) signaling, which can promote epithelial-to-mesenchymal transition and fibrotic tissue remodeling; (2) reduced deferential luminal flow, leading to increased local prostaglandin levels, cellular stress and apoptosis; and (3) decreased circulating insulin-like growth factor 1 (IGF-1), as observed in CFTR KO piglets^45^ and known to be associated with reproductive tract defects in male mice.

So far, no studies have assessed the progression of these abnormalities into adulthood or evaluated spermatogenesis in CF pigs.

## Identifying the most relevant models

Evaluation of male reproductive phenotypes across CF animal models reveals high interspecies variability in the timing, severity and mechanisms underlying reproductive tract abnormalities (Table 1). While >95% of men with CF present with bilateral absence of the vas deferens, no single animal model fully recapitulates the human reproductive phenotype. Overall, CF mouse models, particularly CFTR knock-in or KO models with residual function, fail to reproduce the severity of the male reproductive phenotype observed in humans, as they often remain fertile with intact vas deferens and epididymides. This preservation of the reproductive tract may be partly explained by specific epididymal features in mice, including greater segmentation and a proportionally wider luminal diameter, which may reduce susceptibility to mucus inspissation and secondary obstruction^23^. Another hypothesis is that, similarly to the lungs and pancreas, which do not develop the characteristic CF symptoms observed in humans, alternative chloride channels may be present in the murine reproductive tract, potentially compensating for CFTR loss of function and thereby preventing obstruction and secondary atresia^47^.

CFTR KO mice display age-dependent phenotypes ranging from preserved reproductive tract in young animals to progressive vas deferens atresia, accompanied by spermatogenic alterations. Further investigations, particularly in older CFTR KO mice, are warranted to confirm the phenotype described by Durie et al.^33^, which suggests the occurrence of age-related progressive vas deferens atresia. If validated, this model could provide a valuable tool for studying the mechanisms of secondary vas deferens atresia, although its onset might be delayed compared with humans. Caution is warranted, as most studies have focused on younger animals maintained on mixed backgrounds, making it unclear whether the phenotype reported by Durie et al.^33^ reflects the congenic C57BL/6 background, a later onset of the reproductive phenotype in mice, or both. Knock-in mouse models, meanwhile, do not appear to display any reproductive tract abnormalities.

CFTR KO rats, by contrast, more consistently display absence of bilateral vas deferens with epididymal hypoplasia, more closely mimicking the human phenotype. However, they frequently exhibit more pronounced testicular alterations, including hypospermatogenesis and absence of epididymal sperm, which seem more severe than those currently described in men with CF. Notably, in CFTR KO rats, vas deferens regression does not appear to be consistently associated with intraluminal obstruction, suggesting that mechanisms other than inspissated secretions may be involved^48^. A similar observation has been reported in CF pigs, in which Wolffian structure regression occurs without luminal obstruction or mucus accumulation^45^. While luminal obstruction remains a plausible explanation for vas deferens atresia in men, particularly given the narrower luminal diameter^23^, some evidence suggests that alteration of the proximal epididymal compartment may also contribute to this phenotype. In men with absence of the vas deferens, the caput epididymis is typically present but frequently exhibit pathological changes^49,50^. Human caput epididymal organoid models have shown that CFTR inhibition disrupts epithelial fluid transport and induces a reduction in the size of epididymal structures^49^. Moreover, studies in CFTR-deficient rodent models further indicate that CFTR participates in epithelial proliferation and differentiation through interactions with the Wnt/β-catenin pathway^48^ and tight junction complexes^51^. Loss of CFTR function in these rodent models is associated with altered epithelial differentiation, increased proliferation and cytoarchitectural disorganization of epididymal structures. Together, these findings suggest that intrinsic epithelial dysfunction in proximal epididymal structures may contribute to subsequent vas deferens regression and partial epididymal absence.

Further studies in F508del rats are required to assess the penetrance of vas deferens alterations and their impact on spermatogenesis in adult males. The mechanisms underlying the absence of the vas deferens in CF are probably multifactorial.

Rats could represent a relevant and practical model for studying the CF reproductive phenotype. Housing costs are relatively low, and larger samples sizes than in mice facilitate the visualization and analysis of vas deferens and epididymal structures. While early studies have raised uncertainty regarding the responsiveness of the CFTR-F508del protein in rats to modulator therapies^52^, emerging evidence now suggests that this model is at least partially responsive. Dreano et al.^38^ demonstrated significant restoration of CFTR-dependent chloride transport in CFTR-F508del rat primary nasal epithelial cells using the VX-809 + VX-770 combination. More recently, Reyne et al.^53^ reported in vivo correction of nasal CFTR function following treatment with the triple combination elexacaftor–tezacaftor–ivacaftor in homozygous F508del rats. These findings suggest that rat CFTR-F508del is at least partially responsive to modulator therapies, supporting the utility of this model for evaluating their potential to prevent vas deferens atresia. Further studies are warranted to define the extent and tissue specificity of the modulator response in rats. An alternative approach could be the use of humanized rat models, such as rats expressing hCFTR-G551D, recently reported to respond to ivacaftor. However, the reproductive phenotype in this model has not yet been evaluated^54^.

Among large model animals, ferrets, sheep and pigs consistently display absence of vas deferens but typically show normal testicular histology. However, the extent of epididymal alteration differs across species, ranging from partial to complete absence. No explanation has yet been provided for this variability^39,41–46^. CF is associated with high lethality in these models, mainly due to meconium ileus^21^. In addition, keeping large animals alive until adulthood incurs substantial costs^55^. Consequently, most analyses have focused on the neonatal stage, and no data are currently available regarding the reproductive phenotype in adult males. In pigs, the mechanisms driving Wolffian structures regression seem distinct from human pathophysiology, occurring without mucus accumulation or luminal obstruction. Notably, the CFTR-G551D ferret model has highlighted the potential for prenatal preservation of epididymal and deferential structures through CFTR modulator therapy.

Overall, these findings emphasize the need for complementary use of murine and large-animal models to investigate the mechanisms of reproductive tract abnormalities in CF. The CFTR KO rat model seems to be the most consistent model for the absence of the vas deferens, although the associated testicular alterations warrant caution. Ferrets and pigs reliably mimic the absence of vas deferens observed in humans. However, the mechanisms observed in pigs differ from those described in humans.

Future research should focus on longitudinal evaluation of reproductive outcomes extending into adulthood, particularly in large-animal models for which current data are largely restricted to the neonatal period. Such studies should assess not only reproductive tract anatomy but also spermatogenesis and fertility over time. The effects of aging, which remain largely unexplored in most models, should also be specifically addressed. This issue is becoming increasingly relevant, as highly effective modulator therapy has substantially improved life expectancy in individuals with CF, potentially leading to the emergence of age-related clinical challenges.

In addition, the impact of early and prolonged exposure to CFTR modulators, including in utero and during early childhood, warrants specific investigation. Confirming that pharmacological restoration of CFTR function can prevent vas deferens atresia, and assessing the long-term consequences on testicular development, germ cell maturation and adult fertility, represent critical priorities. Finally, detailed analyses of the pathophysiological mechanisms underlying reproductive tract alterations across species will be necessary to determine to what extent these models faithfully reflect the human disease process.
